# Acquired Brachial Cutaneous Dyschromatosis in a 60-Year-Old Male: A Case Report and Review of the Literature

**DOI:** 10.1155/2014/452720

**Published:** 2014-12-31

**Authors:** Nadia Abidi, Kristen Foering, Joya Sahu

**Affiliations:** Department of Dermatology, Jefferson Medical College, Thomas Jefferson University, 833 Chestnut Street, Suite 740, Philadelphia, PA 19107, USA

## Abstract

Acquired brachial cutaneous dyschromatosis is an acquired pigmentary disorder that has been described in only 20 patients but likely affects many more. This case of a man with acquired brachial cutaneous dyschromatosis is unique as most reports are in women. We report the case of a 60-year-old male who presents with an asymptomatic eruption characterized by hyperpigmented and telangiectatic macules coalescing into patches on the bilateral extensor aspects of the forearms which is consistent clinically and histopathologically with acquired brachial cutaneous dyschromatosis. Given its presence in patients with clinical evidence of chronic sun exposure and its histopathological finding of solar elastosis, acquired brachial cutaneous dyschromatosis is likely a disorder caused by cumulative UV damage. However, a possible association between angiotensin-converting enzyme inhibitors and acquired brachial cutaneous dyschromatosis exists. Further investigation is needed to elucidate both the pathogenesis of the disorder and forms of effective management. Treatment of the disorder should begin with current established treatments for disorders of dyspigmentation.

## 1. Case Report

**Figure 1:**
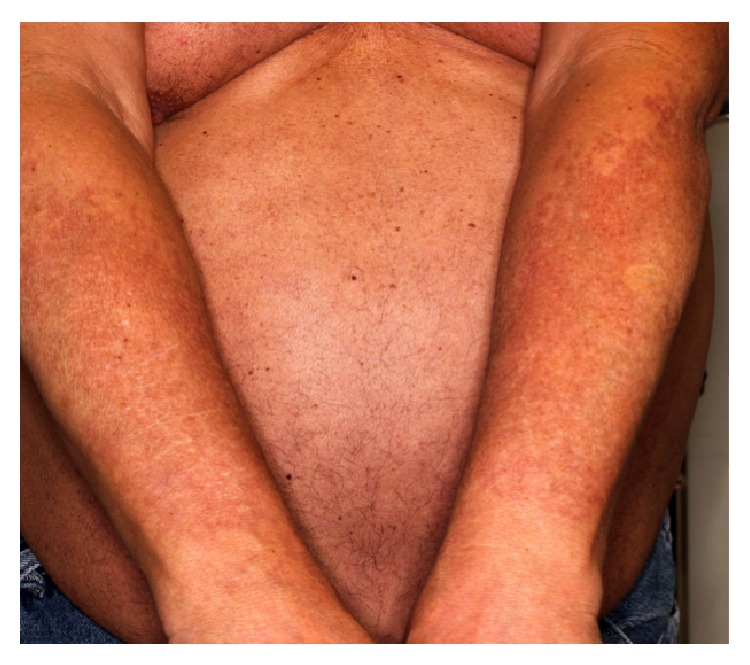
Clinical presentation of acquired brachial cutaneous dyschromatosis. There are irregular hyper- and hypopigmented macules coalescing into large patches on the bilateral dorsal forearms.

**Figure 2:**
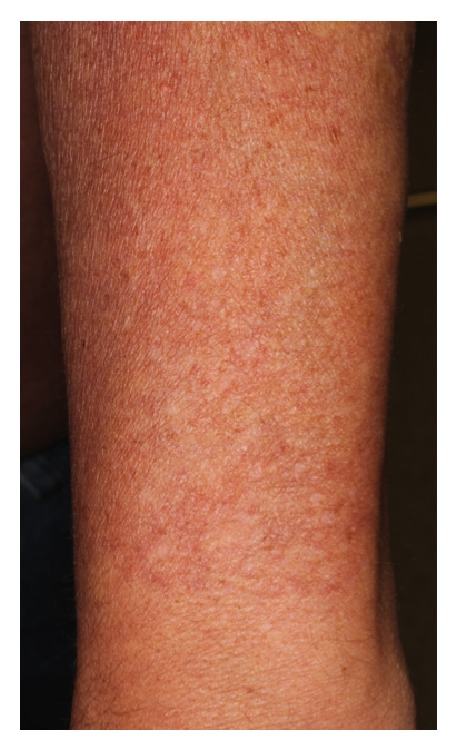
Clinical presentation of acquired brachial cutaneous dyschromatosis. Closer inspection of the left forearm reveals hyperpigmented patches and hypopigmented, slightly depressed, atrophic plaques with prominent telangiectasia. Note the relative sharp demarcation at the distal forearm/wrist.

A 60-year-old male presented for an annual skin examination. Physical exam incidentally revealed two large, well-delineated patches on the bilateral forearms comprised of hyperpigmented, hypopigmented, telangiectatic, and slightly atrophic macules (Figures 1 and 2). Superficial telangiectasias were also present on the neck and anterior chest. The patient was unsure as to when the forearm lesions first appeared but reported a gradual onset and progressive course over several years. On further questioning he denied any associated symptoms. The patient reported a 25-year history of chronic sun exposure secondary to his profession as a fleet service agent handling aircraft cargo transport outdoors. He denied using any consistent form of photoprotection in the form of sunscreen or physical barriers.

The patient is Fitzpatrick skin type III. Past medical history included hypertension, hyperlipidemia, type 2 diabetes mellitus, asthma, and a history of basal cell carcinoma. Current medications include amlodipine/benazepril, which the patient had been taking since his diagnosis of hypertension six years earlier, chlorthalidone, rosuvastatin, metformin, and inhaled mometasone.

**Figure 3:**
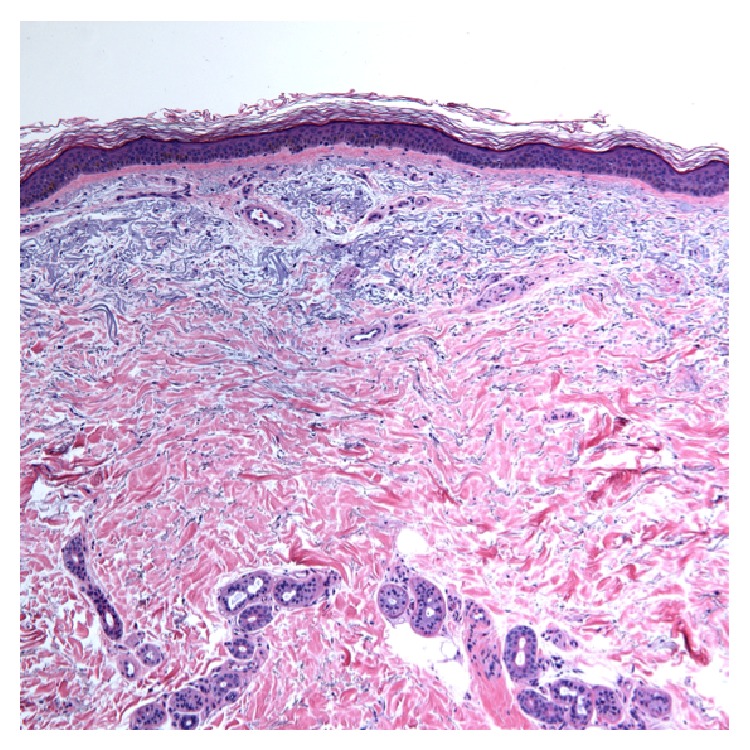
Histopathological examination of acquired brachial cutaneous dyschromatosis. Note the pronounced atrophy of the viable epidermis and papillary dermis, increased telangiectasias, abundant solar elastosis, and scattered melanophages, consistent with ABCD 100x magnification, H&E.

Potassium hydroxide (KOH) preparation on lesion scrapings, performed to rule out underlying fungal infection, was negative. A punch biopsy was obtained from a representative patch on the forearm. Biopsy of the lesion revealed epidermal atrophy with blunting of the rete ridges. There was increased pigmentation of the basal layer without melanin incontinence. There were prominent superficial blood vessels (Figure 3).

## 2. Discussion

Acquired brachial cutaneous dyschromatosis (ABCD) is an acquired disorder of pigmentation of the skin that presents as asymptomatic, gray-brown patches with an irregular geographical border, interspersed with hypopigmented macules on the dorsal aspect of the forearms [1]. It is usually bilateral and distally distributed. On histology, ABCD is characterized by a poikilodermatous-like tissue pattern with epidermal atrophy, increased basal layer pigmentation, solar elastosis, and superficial telangiectases [1–3]. However, unlike poikiloderma, no pigmentary incontinence is seen [1–3]. It has been reported most frequently in middle-aged, postmenopausal women with Fitzpatrick skin types III-IV [1]. Additionally, an association with poikiloderma of Civatte has been found in 45% of cases [1].

The differential diagnosis includes melasma, tinea versicolor, and other disorders of pigmentation. Melasma is comprised of sharply delineated, hyperpigmented macules and patches found primarily on the malar eminences, forehead, upper lip, and mandible of women. It is similar to ABCD histologically in that there is increased basal layer pigmentation, but there is no epidermal atrophy or telangiectasia. Tinea versicolor is characterized by hyper- and hypopigmented macules and patches with fine scale and is commonly found on the neck and trunk in a seborrheic distribution. It is easily diagnosed by microscopic examination of KOH-dissolved scale. Other pigmentary disorders could look similar clinically but they are discernable by histologic examination. Lichen planus pigmentosus (LPP) is characterized by grey to dark brown macules in sun exposed areas such as the face, neck, trunk, and limbs and in sun-protected sites such as the flexural folds [4]. Erythema dyschromicum perstans (EDP) is characterized by asymptomatic, grayish macules involving the trunk and proximal extremities [5]. For both entities, the color is distinctive and different from ABCD and histologically these conditions show interface dermatitis, melanophages, and variability in epidermal change and inflammatory infiltrate [4, 6].

Finally, drugs are known to cause pigmentary disorders and they include nonsteroidal anti-inflammatory drugs, antimalarials, amiodarone, cytotoxic drugs, tetracyclines, heavy metals, and psychotropic drugs [7]. Thorough history distinguished drug-induced hyperpigmentation from ABCD in our patient.

Currently, two hypotheses on the etiopathogenesis of ABCD exist. In its first description in the literature by Rongioletti and Rebora, authors observed a large proportion of their cohort (65%) suffered from hypertension and had been taking antihypertensive drugs for years prior to the onset of the pigmentation—with angiotensin-converting enzyme inhibitors (ACEIs) being the most commonly used [1]. These findings led to the hypothesis of a direct association of ABCD with hypertension and/or with antihypertensive—specifically ACEI—use [1]. Later, Hu et al. disputed over this conclusion, suggesting the association between ABCD and hypertension or antihypertensives as more likely a consequence of the commonality of hypertension and its treatment regimens [2]. Instead, due to its histopathological resemblance to poikiloderma of Civatte, in which epidermal atrophy, hyperpigmentation, and telangiectasias are also seen, Hu et al. suggest the disorder is a manifestation of chronic sun damage—either due to cumulative UV exposure or a pattern produced by drug-induced (possibly ACE-I induced) photosensitivity [2].

Although drug induced pigmentation represents 10–20% of all acquired hyperpigmentation [8], there is currently no data associating ACEIs and cutaneous dyschromia. However, one study reports photosensitivity as an adverse cutaneous reaction to ACEI use [9], therefore potentially backing Hu et al.'s two-hit drug-induced photosensitivity hypothesis. In this case, our patient's long-standing hypertension, use of an ACEI, and chronic sun exposure lend support to either proposed hypothesis.

Though not uncommon according to Rebora and Rongioletti, ABCD is subtle and asymptomatic and thus likely underreported; as such, there is little known about successful treatments. Established treatments for other acquired forms of dyspigmentation, including topical depigmenting agents, chemical peels, and laser treatments, may be considered [10]. To obtain satisfactory cosmetic results, treatment of cutaneous hyperpigmentation often requires the combination of multiple modalities of treatment as well as strict photoprotection. In a 2010 review, authors reported significantly greater improvements in skin pigmentation disorders with treatment using nonablative and ablative fractional photothermolysis (NAFP and AFP) in comparison to treatment with other resurfacing devices [11]. One study utilizing AFP for treatment of poikiloderma of Civatte observed a 65.0% improvement in erythema/telangiectasia and a 66.7% improvement in dyschromia with the average number of 1.4 treatments required for improvement. Due to the histopathological similarities seen in ABCD and poikiloderma of Civatte, we expect that a series of AFP treatments for our patient would result in similar improvement.

In summary, we describe a case of acquired brachial cutaneous dyschromatosis in a 60-year-old male with a history of hypertension, ACEI use, and chronic sun exposure. In most reported cases thus far, individuals have had some evidence of chronic sun exposure either histologically with the presence of solar elastosis or clinically with the presence of poikiloderma of Civatte. Although this most likely indicates a primary sun-exposure component to the etiology of ABCD, pathogenesis via ACEI-induced photosensitivity remains to be investigated. To determine appropriate treatments for ABCD, trials in its management are needed and should be guided by current forms of treatment used for other pigmentation disorders, including topical depigmenting agents, chemical peels, laser treatments, and strict photoprotection.
